# First Principles Study on Electronic Structure and Optical Properties of Ternary GaAs:Bi Alloy

**DOI:** 10.3390/ma5122486

**Published:** 2012-11-26

**Authors:** Lifei Yu, Dechun Li, Shengzhi Zhao, Guiqiu Li, Kejian Yang

**Affiliations:** School of Information Science and Engineering, Shandong University, Jinan 250100, China; E-Mails: yulifei1234@126.com (L.Y.); shengzhi_zhao@sdu.edu.cn (S.Z.); gqiuli@sdu.edu.cn (G.L.); k.j.yang@sdu.edu.cn (K.Y.)

**Keywords:** band structure, partial density of states, optical properties, first principles

## Abstract

The electronic structure and optical properties of ternary GaAs:Bi alloy are investigated by first principles calculations. It is found that the band gap of GaAs_1-x_Bi_x_ decreases monotonously with the increasing of Bi concentration, resulting in the fundamental absorption edge and main absorption peaks of GaAs_1-x_Bi_x_ shifting toward lower energy with the increase of the Bi content. The optical constants of GaAs_1-x_Bi_x_, such as the optical absorption coefficient, refractive index, extinction coefficient and optical conductivity, are greater than those of pure GaAs when x > 3.1%, but less than those of pure GaAs when x < 3.1%, which is primarily decided by the intraband level repulsions between Bi-induced states and host states on the valence bands; the contribution of Bi-6*s*, Bi-6*p* orbitals and Ga-4*p*, Ga-4*s* orbitals on conduction bands is also crucial. Bi doping plays an important role in the modulation of the static dielectric constant and the static refractive index. These results suggest a promising application of GaAs_1-x_Bi_x_ alloy as a semiconductor saturable absorber.

## 1. Introduction

In recent years, there is much interest in the characterization of GaAs crystal because of its potential optoelectronics applications in the various fields. For instance, GaAs has caused widespread concern as a semiconductor saturable absorber for its advantages of stable photochemical property, good thermal conductivity, non-degradability and high damage threshold [1,2,3,4]. GaAs-based solar cells also have many advantages, such as high photoelectric conversion efficiency, fine radiation resistance and good performance at high temperatures, *etc*. [5,6]. As science and technology develops, people always expect to broaden the applications of GaAs. Doping other elements is one of the most commonly used methods, and is performed by replacing a small amount of anion species in GaAs with isovalent impurities, such as N, P, Sb and Bi. The ternary alloy GaAs_1-x_Bi_x_ has been successfully grown by metal organic chemical vapor deposition (MOCVD) [7,8], and quite recently by molecular beam epitaxy (MBE) [9,10,11,12,13,14]. Existing research shows that GaAs_1−x_Bi_x_ alloy are strong potential candidate materials for long wavelengths emitters and detectors as well as spintronic-related devices [15,16].

On the theoretical side, the calculated electronic properties of GaAs_1-x_Bi_x_ show that Bi doping can reduce the band gap of GaAs, and the effect is more significant than alloying other III-V compound semiconductors at the same concentration [17]. Recent research of the GaAsBi system is mainly concerned with the lattice constant, alloy formation energy, energy level position and composition evolution of the system [17,18,19], however, the optical properties have not yet been reported in detail, and the relationship between optical properties and electronic structure are scarcely investigated. It is important to study theoretically the electronic structure and optical properties of GaAs_1-x_Bi_x_ alloy, which can be applied in more tech areas. For instance, it can make GaAs_1−x_Bi_x_ a promising new semiconductor saturable absorber that has a very high possibility to be used in all-solid-state Q-switched and mode-locked lasers.

In this paper, GaAs_1−x_Bi_x_ crystal with different concentrations of Bi atoms (x = 0, 2.1%, 2.5%, 3.1%, 6.3%, 12.5%), were investigated by the first principles calculations based on the plane wave and pseudo-potential approach. The electronic structure including band structure and partial density of states were obtained, and the optical properties, such as the complex dielectric function, absorption coefficient, complex refractive index, extinction coefficient and optical conductivity, were also studied. It is found that the band gap of GaAs_1−x_Bi_x_ decreases monotonously with the increase of Bi concentration, resulting in the fundamental absorption edge and main absorption peaks of a GaAs_1-x_Bi_x_ shift toward lower energy with the increase of Bi content, and Bi doping plays an important role in the modulation of the static dielectric constant and the static refractive index, which is primarily decided by the intraband level repulsions between Bi-induced states and host states on the valence bands, and the contribution of Bi-related impurity states of conduction bands is also crucial.

## 2. Simulation Method and Theoretical Description

### 2.1. Simulation Method and Model

Cambridge serial total energy package (CASTEP) [20], an *ab initio* pseudo-potential method based on DFT, was used in this paper. GGA with PBE [21] parameterization was adopted to describe the exchange–correlation interaction. Ultrasoft pseudo-potentials [22] were applied to model the electron–ion interaction. Face-centered cubic cell was selected as the computational model, corresponding to x = 12.5%, 6.3%, 3.1%, 2.5%, 2.1% and 0, super-cells of 1 × 1 × 2, 1 × 2 × 2, 2 × 2 × 2, 1 × 2 × 5, 2 × 2 × 3, 2 × 2 × 2 were adopted, in which one As atom was replaced by Bi atom. 2 × 4 × 4, 2 × 2 × 4, 2 × 2 × 2, 4 × 2 × 1, 2 × 2 × 1, 2 × 2 × 2 K-point Monkhorst-Pack mesh were applied to the bulks. The lattice parameters were experimental values (a = b = c = 0.5653 nm), and the entire system was electronic neutrality. As the Bi concentration increased, the lattice parameters experienced minor changes. In this paper, we mainly focus on the electronic structure and the optical properties of GaAs_1−x_Bi_x_, and have refrained from including a discussion of the minor changes of lattice parameters. The BFGS algorithm [23,24] was chosen for the geometry optimization of the super-cells. The energy cut-off for the plane wave basis was chosen as 330 eV for the electronic structure calculation and optical properties calculation. The tolerances were set as follows: 1 × 10^−6^ eV/atom for the total energy, 0.5 eV/nm for maximum force, 0.1 GPa for pressure and 0.0001 nm for displacement. SO interaction was not considered in this calculation, because our calculation was done in the independent-particle approximation. Furthermore, the band structure and the density of states of GaAs_1−x_Bi_x_ did not show any difference with or without SO interaction. Finally, we overcame the well-known problem that DFT underestimates the band gap. Thus, in our calculation, the scissors operator [25,26] correction was used to improve the calculation accuracy of the electronic structure and the optical properties. Comparing with the GaAs band gap of 1.42 eV, we adopted 1.0 eV as the value of the scissors operator.

### 2.2. Theoretical Description of Optical Properties

The dielectric function describes the linear response of the system to electromagnetic radiation, and it dominates the behavior of electromagnetic wave propagation in the medium. It is closely related to the electron–photon interaction, as a bridge that connects the physical process of interband transition with the solid electronic structure, we can easily use it to obtain other spectrums. The imaginary component of the dielectric function $\varepsilon_{2}(\omega )$ , which can be deduced from the definition of direct transition probability, mainly characterizes the electron transition from the occupied states to unoccupied states. Within the linear response range, solid macro-optical response function can usually be described by the complex permittivity: $\varepsilon \left(\omega \right)=\varepsilon_{1}\left(\omega \right)+i\varepsilon_{2}\left(\omega \right)$ or complex refractive index: N(ω)=n(ω)+ik(ω)

(1) $$\varepsilon_{1}=n^{2}-k^{2}$$

(2) $$\varepsilon_{2}=2nk$$

By the Kramers–Kronig relationship, $\varepsilon_{1}(\omega )$ can be obtained by integration over a fairly wide frequency range using a differential coefficient of $\varepsilon_{2}(\omega )$ . Then the absorption coefficient and the optical conductivity can be inferred [27].

(3) $$\varepsilon_{2}=\frac{C_{1}}{\omega^{2}}\sum_{V.C}\int_{BZ}d^{3}K\frac{2}{\left(2\pi \right)}{\left|e\cdot M_{CV}\left(K\right)\right|}^{2}\delta \left[E_{C}\left(K\right)-E_{V}\left(K\right)-\text{ℏ}\omega \right]$$

(4) $$\varepsilon_{1}=1+C_{2}\sum_{V.C}\int_{BZ}d^{3}K\frac{2}{\left(2\pi \right)}\frac{{\left|e\cdot M_{CV}\left(K\right)\right|}^{2}}{\left[E_{c}\left(K\right)-E_{V}\left(K\right)\right]}\frac{{\text{ℏ}}^{3}}{{\left[E_{C}\left(K\right)-E_{V}\left(K\right)\right]}^{2}-{\text{ℏ}}^{2}\omega^{2}}$$

(5) $$I\left(\omega \right)=\sqrt{2}\left(\omega \right){\left[\sqrt{\varepsilon_{1}{\left(\omega \right)}^{2}-\varepsilon_{2}{\left(\omega \right)}^{2}}-\varepsilon_{1}\left(\omega \right)\right]}^{\frac{1}{2}}$$

(6) $$\sigma_{r}=\varepsilon_{0}\omega \varepsilon_{1}(\omega )$$

Where *C* and *V* are the conduction band and valence band, respectively, *BZ* is the first Brillouin zone, *K* is the electron wave vector, ${\left|e\cdot M_{CV}(k)\right|}^{2}$ is momentum transition matrix element, ω is the angular frequency, *C_1_* and *C_2_* are constants, $E_{C}(K)$ and $E_{V}(K)$ are the intrinsic energy level of the conduction band and valence band, respectively. *I*( ω ) is the absorption coefficient, $\varepsilon_{0}$ is the static dielectric constant, and $\sigma_{r}$ is the optical conductivity. The above relationships are the theoretical basis of the optical properties of crystal, which reflect the luminescence mechanism of the spectrum.

## 3. Results and Discussion

### 3.1. Electronic Structure

**Figure 1:**
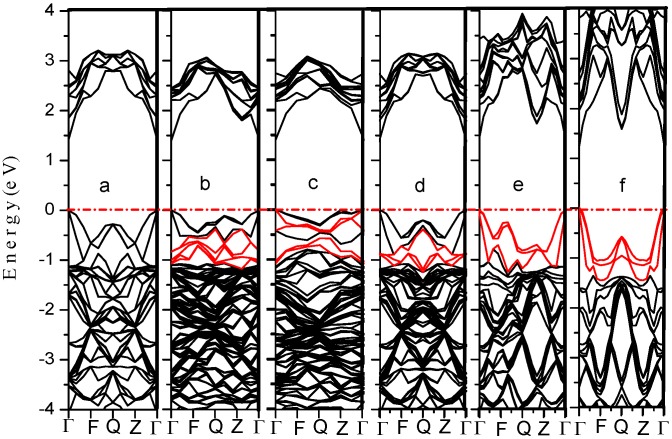
The band structure of GaAs_1−x_Bi_x_ (**a**) x = 0; (**b**) x = 2.1%; (**c**) x = 2.5%; (**d**) x = 3.1%; (**e**) x = 6.3%; (**f**) x = 12.5%.

Figure 1 shows the band structure of GaAs_1−x_Bi_x_ with different Bi concentrations, and the coordinates of the special *K*-points in Figure 1 are: Γ (0.000, 0.000, 0.000), F (0.000, 0.500, 0.000), Q (0.000, 0.500, 0.500), Z (0.000, 0.000, 0.500), which are the high-symmetry points of Face-centered cubic cell. We can see that GaAs_1−x_Bi_x_ is a typical direct band gap semiconductor, and the band gap shows a downward trend as the Bi concentration increases. The result has been given in Table 1, which is close to the previous reported results in [28], as the band gaps in [28] calculated with the Hybrid function method are relatively accurate. Although the calculated values for the band gap do not agree with other reported ones [16,19,29,30](due to the systematic error of those calculations), this method is still able to provide valuable information about the material’s other characteristics, such as the optical properties. New defect bands related to the doped Bi atoms are found in valence band (highlighted in red), which will be referred to as impurity states.

**Table 1:** Band gap *E_g_* (eV) of GaAs_1−x_Bi_x_ with different Bi content.

Name	The value					
*X*	0	2.1%	2.5%	3.1%	6.3%	12.5%
*E_g_*	1.419	1.390	1.385	1.362	1.342	1.208

**Figure 2:**
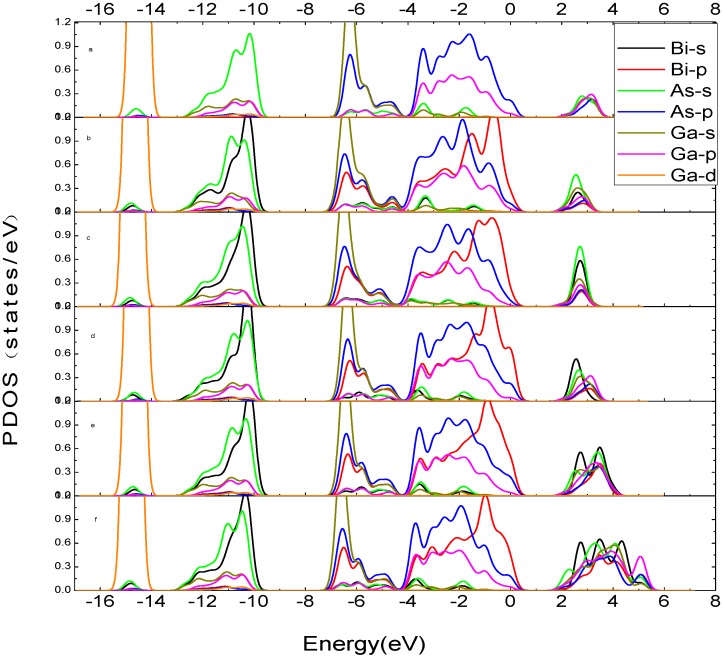
The PDOS of GaAs_1-x_Bi_x_ (**a**) x = 0; (**b**) x = 2.1%; (**c**) x = 2.5%; (**d**) x = 3.1%; (**e**) x = 6.3%; (**f**) x = 12.5%.

Figure 2 shows the partial density of states (PDOS) of GaAs_1−x_Bi_x_ with different Bi concentrations. As shown in Figure 2, the valence bands are attributed to As-4*p* and Bi-6*p* orbitals, and the conduction bands depend on Ga-4*s*, Ga-4*p*, Bi-6*s* and As-4*s* orbitals. The valence bands are composed of the lower valence bands which locate from –16 eV to –9 eV, and the upper valence bands which locate from −7 eV to 0 eV. The lower valence bands are mainly attributed to Ga-3*d*, Bi-6*s* and As-4*s* orbitals. There is a sharp peak of Ga-3*d* orbitals in –14.83 eV, which forms strong localized states, and its intensity is much larger than the As-4*s* orbital and Bi-6*s* orbital. The upper valence bands can also be divided into two parts, from −7.20 eV to −5.14 eV, mainly lying on Ga-4*s*, As-4*p* and Bi-6*p* orbitals, while near the Fermi energy level (−4.43 eV to 0 eV), the valence bands mainly rest with As-4*p*, Bi-6*p* and Ga-4*p* orbitals. The top of the valence bands are decided by As-4*p* and Bi-6*p* orbitals. For conduction bands, the hybrid orbitals which are formed by 4*s*/4*p* orbitals of As and Ga atoms are crucial; 6*s*/6*p* hybrid orbitals of Bi atoms are also essential, and it can be seen that the *sp* hybrid orbitals of conduction bands of Bi atoms and Ga atoms have been enhanced when the doping concentration is x > 3.1% from Figure 2.

Therefore, the valence bands of GaAs_1−x_Bi_x_ mainly depend on Ga-4*p*, As-4*p* and Bi-6*p* orbitals; the conduction bands of GaAs_1−x_Bi_x_ are mainly attributed to 4*s*/4*p* orbitals of Ga and As and 6*s*/6*p* orbitals of Bi.

### 3.2. Optical Properties

The optical properties of GaAs_1−x_Bi_x_ mainly refer to the dielectric function, absorption coefficient, the complex refractive index and optical conductivity, which are primarily decided by the bands near the Fermi energy level, the concentration, and the mobility of carrier.

#### 3.2.1. Complex Dielectric Function

**Figure 3:**
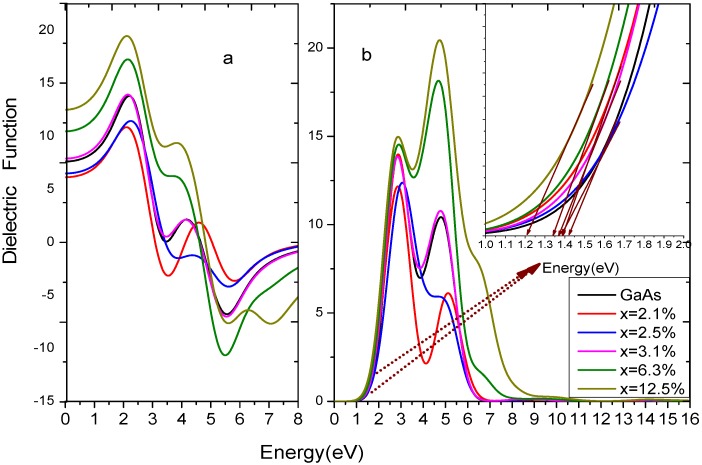
The complex dielectric function of GaAs_1−x_Bi_x_. (**a**) the real component; (**b**) the imaginary component.

Figure 3a,b show the real and imaginary component of the dielectric function, which can be obtained by Equations 3 and 4. In low-energy areas, the real component of the dielectric function increases with the photon energy, and gets maximum value when the energy reaches about 2.0 eV, because $\varepsilon_{1}$ can get the maximum value and the minimum value, respectively, at the maximum slope of $\varepsilon_{2}$ when in rise or decline. They reach the second peak when the energy is about 4.0 eV, then they decrease with the increase of photon energy. When x is 0, 2.1%, 2.5%, 3.1%, 6.3%, 12.5%, the static dielectric constant has been calculated. For GaAs, its dielectric constant is $\varepsilon_{1}\left(\omega \right)$ = 13.34 when the photon energy is 2.00 eV, which is close to the calculation 14.99 in [31]. As shown in Table 2, the static dielectric constants change with an increase of doping concentration. x = 3.1% is a demarcation line, the dielectric function diagram of GaAs_1-x_Bi_x_ at this content is very similar to the pure GaAs, so we define high doping as x > 3.1% and low doping a x < 3.1%. The static dielectric constants are quite different in high or low doping, so Bi doping play an important role in modulation of the static dielectric constant [32].

**Table 2:** The static dielectric constant *ε_0_* (F/m) of GaAs_1-x_Bi_x_ with different Bi content.

Name	The value					
*X*	0	2.1%	2.5%	3.1%	6.3%	12.5%
*ε_0_*	7.63	6.15	6.50	7.90	10.47	12.51

From the inset of Figure 3b, the fundamental absorption edge of GaAs_1−x_Bi_x_ can be obtained. It can be seen that the results are in good agreement with the direct transition [28]. For the absorption edge of pure GaAs, it is due to the transitions from As-4*p* orbitals to Ga-4*s* orbital, while for GaAs_1-x_Bi_x_, the transitions are from As-4*p* and Bi-6*p* orbitals to Ga-4*s* orbital. With the increasing of photon energy, there are two main peaks for $\varepsilon_{2}(\omega )$ of GaAs_1-x_Bi_x_, which may depend on the density of states and the electronic transition. The first peaks locate at 2.8 eV–3.1 eV, and when x is 0, 2.1%, 2.5%, 3.1%, 6.3%, or 12.5%, the corresponding photon energy is 2.87 eV, 2.85 eV, 3.06 eV, 2.85 eV, 2.90 eV, 2.87 eV, respectively. The pure GaAs, rest with the interband transitions from As-4*p* orbitals to Ga-4*s* orbital, while 2.1%, 2.5%, 3.1%, 6.3%, and 12.5% correspond to the interband transition from Bi-6*p* orbitals and As-4*p* orbitals to Bi-6*s* orbital. The second dielectric peaks locate at 4 eV–5 eV, and when x is 0, 2.1%, 2.5%, 3.1%, 6.3%, or 12.5%, the corresponding photon energy is 4.80 eV, 5.12 eV, 5.06 eV, 4.78 eV, 4.69 eV, 4.73 eV, respectively. For the pure GaAs, it depends on the interband transitions from As-4*p* orbitals to Ga-4*s* and As-4*s* orbitals, while for x = 2.1%, 2.5%, 3.1%, 6.3%, 12.5%, corresponding to the interband transition from As-4*p* orbitals on valence bands to Bi-6*s* orbital, As-4*s* orbital and Ga 4*s*/4*p* orbitals on conduction bands. In high doping, almost all the peaks show obvious redshifts, owing to the valence bands broadening, which are ascribed to intraband level repulsions between Bi-induced states and host states [28].

#### 3.2.2. Absorption Spectra

**Figure 4:**
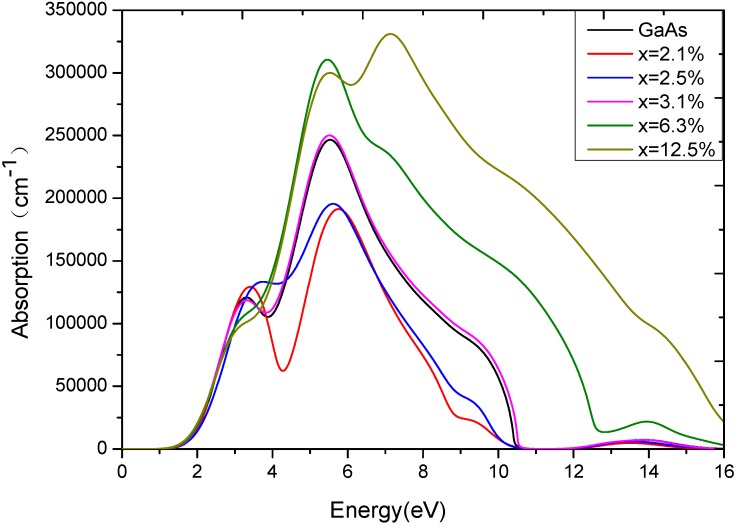
The absorption spectrum of GaAs_1-x_Bi_x_.

The light absorption coefficient expresses the percentage of light intensity attenuation per units of distance traveling in the medium. The absorption spectrum of GaAs_1−x_Bi_x_ is obtained by Equation 5. Figure 4 shows that GaAs with different Bi content all have peaks between 3.5 eV and 5.5 eV, which is identical with the result of the imaginary component of dielectric function $\varepsilon_{2}(\omega )$ . There is a crosspoint at about 3.6 eV in Figure 4. When the photon energy is less than 3.6 eV, the absorption coefficient of GaAs_1-x_Bi_x_ in low doping is greater than that of pure GaAs and high doped GaAs_1-x_Bi_x_. However, when the photon energy is more than 3.6 eV, the absorption coefficient shows a completely opposite phenomenon: the high doped GaAs_1−x_Bi_x_ has the strongest absorption coefficient. How does this happen? It is mainly attributed to the intraband level repulsions between Bi-induced states and host states. Investigating the band structure of GaAs_1−x_Bi_x_ in Figure 1 and the PDOS of GaAs_1−x_Bi_x_ in Figure 2, we discover that Bi-6p and As-4p orbital occupy the position of about −1 eV below the Fermi energy level together in low doping, as the repulsive force is very weak. The repulsive force become so strong, makingBi-6p and As-4p orbital separated in high doping, leaving only the Bi-6p orbital still occupying the −1 eV position on the valence band. Therefore, the low doped GaAs_1-x_Bi_x_ has the main occupancy of states at the area near −1 eV on the valence bands, and the high doped GaAs_1−x_Bi_x_ has the main occupancy of the states in the range from −3 eV to −1 eV on the valence bands. According to the interband transitions analysis in the dielectric function, the probability of optical transition of low doped GaAs_1-x_Bi_x_ is more than that of high doped GaAs_1−x_Bi_x_ when the photon energy is less than 3.6 eV, while the results reverse when the photon energy is more than 3.6 eV. When x = 3.1%, Bi-6p and As-4p orbital of GaAs_0.969_Bi_0.031_ occupy a particular position because of the repulsive force, what makes the probability of interband transition of GaAs_0.969_Bi_0.031_ shows similar to pure GaAs, and the probability exactly located between the high doped and the low doped GaAs_1−x_Bi_x_. Within the energy range of 5.46 eV to 5.90 eV, Figure 4 clearly shows that the absorption coefficient of GaAs_1−x_Bi_x_ varies widely when Bi content changes from low to high. High doping GaAs, whose absorption coefficient is much higher than those of pure GaAs, their difference is about 5 × 10^4^ cm^−1^, while the absorption coefficient of pure GaAs is much higher than those of low doped GaAs, and their difference is also about 5 × 10 cm^−1^. Considering the analysis above, we find that in the area from −2 eV to −1 eV on the valence bands, both the high doped GaAs_1-x_Bi_x_ and the low doped GaAs_1-x_Bi_x,_ are mainly attributed to the As-4p orbital, but the density of As-4p orbital is different. Furthermore, the conduction bands of GaAs_1−x_Bi_x_ vary considerably as *x* value changes. When in low doping, the conduction bands mainly depend on Bi-6*s* and As-4*s* orbital and 4*s*/4*p* orbitals of Ga, while the *p*-orbitals of Bi and As atoms contribute little to the conduction bands. For these reasons, the conduction bands become weaker in low doping, and the optical transition probability from valence bands to conduction bands also decrease. When in high doping, not only the *s*-orbital but also the *p*-orbitals of Bi and As atoms greatly contribute to the conduction bands, thus enhancing significantly the probability of optical transition from valence bands to conduction bands. The light absorption rate is also increased, which reflects the good saturable absorption property of GaAs in high doping in the energy range of 5.46 Ev–5.90 eV. The main absorption peaks and the absorption edge of GaAs_1−x_Bi_x_ show redshifts compared with the corresponding peaks of GaAs, coinciding with the result of the imaginary component of dielectric function.

#### 3.2.3. Complex Refractive Index

**Figure 5:**
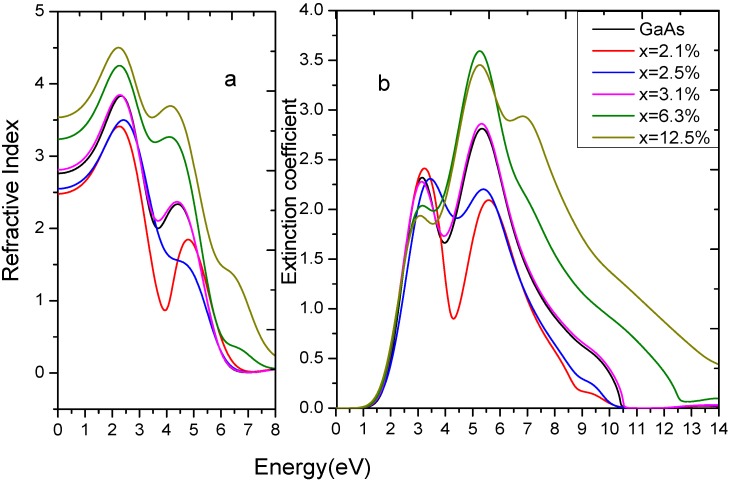
The refractive index and the extinction coefficient of GaAs_1−x_Bi_x_

From the relationship of the complex refractive index and the complex dielectric function, shown in Equations 1 and 2, we can obtain the complex refractive index of GaAs_1−x_Bi_x_. The refractive index and the extinction coefficient are shown in Figure 5 (5a is the refractive index and 5b is the extinction coefficient) and Table 3 shows the static refractive index. For GaAs, its refractive index is n = 3.16 when the photon energy is 1.43 eV, which is very close to the experimental values 3.59 [33]. It can be seen that the static refractive index of GaAs_1−x_Bi_x_ exhibits different changing trends with the increase of Bi-doped concentration. The constant is significantly greater than those of pure GaAs in high doping, while it shows the opposite result in low doping. Meanwhile, the static refractive index increases with the increasing of doped concentration, which means that Bi doping plays an important role in modulation of the static refractive index. This is consistent with the static dielectric constant. The main peaks of *n* are in the energy range of 1.90 eV–4.80 eV. The refractive index began to decline when the photon energy was larger than 4.80 eV. According to $n^{2}(\omega )-k^{2}(\omega )=\varepsilon_{1}(\omega )$ , the valley of $\varepsilon_{1}(\omega )$ is corresponding to the peak of k(ω) in this frequency range. Figure 5 shows that the extinction coefficients achieve the maximum peak when the photon energy is about 5.50 eV, and the peaks of *k* shift toward lower energy with increasing Bi content, which are relevant to the result of $\varepsilon_{2}(\omega )$ .

**Table 3:** The static refractive index of GaAs_1-x_Bi_x_ with different Bi content.

Name	The value					
*X*	0	2.1%	2.5%	3.1%	6.3%	12.5%
*n(0)*	2.76	2.48	2.55	2.81	3.24	3.54

#### 3.2.4. Optical Conductivity

**Figure 6:**
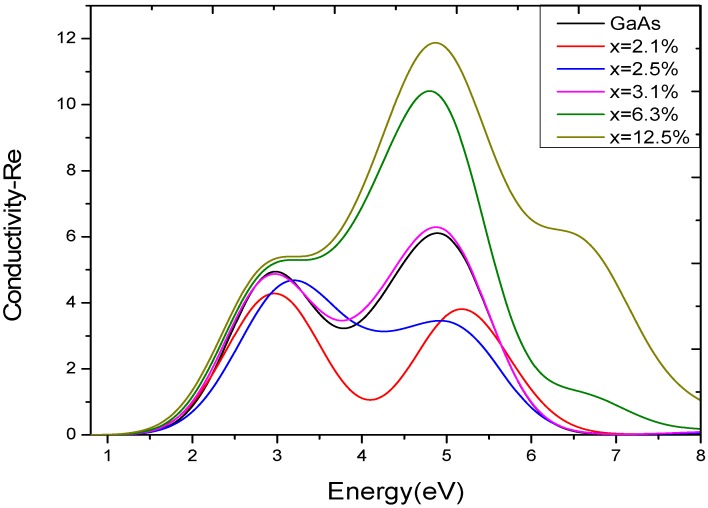
The real component of optical conductivity of GaAs_1−x_Bi_x_.

Semiconductor optical conductivity is the change in conductivity caused by illumination, either an increase or a decrease. The photoconductive effect is the physical basis of optoelectronic applications of semiconductors. It can be obtained by Equation 6. Figure 6 is the real component of the optical conductivity. When the photon energy is less than 1 eV, the optical conductivity is 0. For doping concentration *x* equals to 0, 2.1%, 2.5%, 3.1%, 6.3%, or 12.5%, the corresponding optical conductivity demonstrates high doping and low doping two different changing trends. As Figure 2 shows, when GaAs_1−x_Bi_x_ is in high doping, the conduction bands near the Fermi energy level have introduced a large number of Bi-6*s* electrons and a small number of Bi-6*p* electrons, which significantly enhanced the conductivity of the system. When the energy is about 3 eV, there is a peak, and the conductivity reaches the maximum peak when the energy is near 5 eV. The peaks of conductivity shift toward lower energy with the increasing of Bi-doped concentration, which corroborates the result of $\varepsilon_{2}(\omega )$ .

## 4. Conclusions

In summary, *via* first principles calculations, we have investigated the electronic structure and optical properties of GaAs_1-x_Bi_x_ by tuning Bi concentration. It is found that the band gap of GaAs_1−x_Bi_x_ decreases monotonously with increasing of Bi concentration, resulting in the fundamental absorption edge and main absorption peaks of the GaAs_1−x_Bi_x_ shift toward lower energy with the increase of Bi content. The optical constants, including dielectric function, absorption coefficient, complex refractive index and optical conductivity, are greater than those of pure GaAs when x > 3.1%, but less than those of pure GaAs when x < 3.1%, which is primarily decided by the intraband level repulsions between Bi-induced states and host states on the valence bands, and the contribution of Bi-6*s*, Bi-6*p* orbitals and Ga-4*p*, Ga-4*s* orbitals on conduction bands. Bi doping plays an important role in the modulation of the static dielectric constant and the static refractive index. The band gap narrowing effect and the special optical properties suggest that the GaAs_1-x_Bi_x_ alloy is a promising new semiconductor saturable absorber for the future.
